# Editorial: Upping the heat: capsaicin for human health

**DOI:** 10.3389/fnut.2025.1665722

**Published:** 2025-07-29

**Authors:** Ajay Kumar, Guiomar Melgar-Lalanne, Hércules Rezende Freitas, Ricardo Diego Duarte Galhardo de Albuquerque, Robson Xavier Faria

**Affiliations:** ^1^Department of Plant Science, School of Biological Sciences, Central University of Kerala, Kasaragod, India; ^2^Instituto de Ciencias Básicas, Universidad Veracruzana, Xalapa, Mexico; ^3^Departamento de Ciências Médicas Integradas, Faculdade de Ciências Médicas, Universidade do Estado do Rio de Janeiro, Rio de Janeiro, Brazil; ^4^Facultad de Farmácia, National University of Trujillo, Trujillo, Peru; ^5^Laboratório de Avaliação e Promoção de Saúde Ambiental, Oswaldo Cruz Institute, Oswaldo Cruz Foundation (Fiocruz), Rio de Janeiro, Brazil

**Keywords:** the transient receptor potential vanilloid 1 (TRPV1), obesity, ionic channel, chili pepper, pain

Capsaicin, the pungent alkaloid derived from chili peppers, has long intrigued scientists and clinicians for its complex and often paradoxical physiological effects. Historically studied for its sensory and nociceptive properties via the transient receptor potential vanilloid 1 (TRPV1) channel, recent advances have expanded our understanding of capsaicin far beyond its role as a TRPV1 agonist (1). From its application in neuroendocrine regulation and metabolic control to its emerging anticancer potential and systemic effects through membrane modulation, capsaicin occupies a unique niche in translational research (2, 3). This Research Topic, *Upping the Heat: Capsaicin for Human Health*, gathers four multidisciplinary contributions that collectively explore capsaicin's biochemical versatility, molecular targets, and implications for human physiology and disease.

In the mini-review “*Role of TRPV1 in neuroendocrine regulation: a potential target against obesity?*”, Wang et al. address one of the most pressing global health challenges: obesity. Given that obesity is projected to affect over 4 billion individuals by 2035, novel molecular targets for metabolic regulation are urgently needed. TRPV1, a nonselective cation channel initially recognized for its role in pain signaling, is increasingly recognized as a key modulator of energy balance, feeding behavior, adipose tissue thermogenesis, and mitochondrial function. The authors synthesize recent findings demonstrating that TRPV1 activation influences neuropeptide signaling (e.g., CGRP, substance P, α-MSH), upregulates genes associated with satiety (such as *PYY, BDNF*, and *CARTPT*), and downregulates orexigenic signals, including *NPY* and *GHSR*. Interestingly, bidirectional control of the channel across the central and peripheral systems suggests a neuroendocrine loop in which TRPV1 integrates vagal afferent input, sympathetic output, and local adipocyte signaling. These mechanisms support TRPV1 as a viable target for pharmacological modulation aimed at reducing obesity and improving metabolic outcomes.

While the aforementioned review foregrounds TRPV1, the mini-review “*Capsaicin: beyond TRPV1*” by Juárez-Contreras et al. broadens the scope to explore TRPV1-independent actions of capsaicin. The amphiphilic nature of capsaicin allows it to integrate into lipid bilayers, altering membrane fluidity and curvature, which in turn modulates ion channel activity and membrane protein function independently of receptor-ligand binding. This biophysical property enables capsaicin to influence various voltage-gated channels, including Nav1.5 in cardiac tissues, and ligand-gated channels such as ASICs and P2X receptors. This review highlights how these membrane-level interactions can either enhance or suppress ion flux depending on the capsaicin concentration and membrane context, emphasizing the dose-dependent duality of physiological outcomes. Moreover, the article explores unclassical targets such as tumor-associated carbonic anhydrases (CA IX and CA XII), where capsaicin acts as a selective inhibitor, suggesting anticancer mechanisms independent of TRPV1. This recontextualization of capsaicin as a membrane-active compound opens new avenues for investigating its systemic effects on nonneuronal tissues.

A direct extension of this conceptual shift was presented in the original research article “*Preparation, characterization, and anticancer effect of Capsaicin-functionalized selenium nanoparticles*” by Tang et al.. This work exemplifies a translational leap by engineering capsaicin onto selenium nanoparticles (Cap@SeNPs) to enhance both bioavailability and therapeutic potential. Selenium, a trace element essential for antioxidant defense and immune regulation, is limited in therapeutic contexts by its instability and narrow therapeutic index. Functionalizing selenium nanoparticles with capsaicin not only stabilizes the nanostructures but also synergizes two bioactive compounds with distinct mechanisms. In HepG2 liver cancer cells, Cap@SeNPs induced apoptosis through ROS generation, mitochondrial membrane depolarization, nuclear condensation, and caspase activation. Importantly, the hybrid nanoparticles exhibited superior efficacy over either compound alone. This research illustrates how the chemical versatility of capsaicin can be harnessed to improve drug delivery and therapeutic specificity, particularly in oncology.

However, while these molecular and cellular findings paint an optimistic picture, the broader epidemiological impact of capsaicin consumption remains contested. In their cross-sectional analysis “*Does chili pepper consumption affect BMI and obesity risk?*”, Liu et al. provide a counterpoint to mechanistic enthusiasm by examining real-world associations in the U.S. population via NHANES data (2003–2006). Interestingly, the study revealed that higher chili pepper consumption frequency was associated with increased BMI and obesity incidence, particularly in females and individuals over 60 years of age. These findings contrast with those of preclinical and mechanistic studies suggesting the anti-obesogenic effects of capsaicin. Several explanations may reconcile this discrepancy, including differences in chili pepper variety, spiciness, or intake amount, as well as compensatory energy intake, capsaicin tolerance, or the form in which capsaicin is consumed (e.g., fresh chili vs. purified extract). Moreover, cultural and geographical variability in chili consumption complicates interpretation (4), underscoring the need for longitudinal and interventional studies that control for confounders.

Together, these four contributions demonstrate the dualistic nature of capsaicin, both as a tool and a variable, in human health research. Capsaicin has emerged as a bioactive compound with therapeutic potential across metabolic, oncologic, and cardiovascular domains, acting through TRPV1-dependent and TRPV1-independent mechanisms. On the other hand, its epidemiological footprint remains ambiguous, highlighting the need to contextualize molecular mechanisms within population-level behaviors and exposures. Bridging this gap will require integrative research that combines cellular, systemic, and population health methodologies.

This Research Topic thus serves as a timely reflection of capsaicin's evolving scientific narrative. Once studied primarily as a neurotoxin and flavoring agent, it is now positioned at the intersection of nutrition, pharmacology, and bioengineering. As the field moves forward, future studies will need to address several open questions: Can TRPV1 activation be safely targeted without triggering nociceptive side effects? How can capsaicin's pleiotropic actions be modulated for tissue specificity? What is the optimal dose and delivery system for clinical applications? And importantly, how do individual variability, sex, age, and dietary context influence capsaicin's efficacy and safety?

As editors, we hope this Research Topic stimulates interdisciplinary dialogue and encourages further investigation into capsaicin's multifaceted roles in human health. Whether as a signaling molecule, membrane modulator, or drug delivery agent, capsaicin undoubtedly “ups the heat” in biomedical research.
